# Tissue-specific extracellular matrix for the larger-scaled expansion of spinal cord organoids

**DOI:** 10.1016/j.mtbio.2025.101561

**Published:** 2025-02-11

**Authors:** Yanjun Guan, Zhibo Jia, Xing Xiong, Ruichao He, Yiben Ouyang, Haolin Liu, Lijing Liang, Xiaoran Meng, Ranran Zhang, Congcong Guan, Sice Wang, Dongdong Li, Yuhui Cui, Jun Bai, Jinjuan Zhao, Haoye Meng, Jiang Peng, Yu Wang

**Affiliations:** a Institute of Orthopedics, The Fourth Medical Center of Chinese PLA General Hospital, Beijing Key Lab of Regenerative Medicine in Orthopedics, Key Laboratory of Musculoskeletal Trauma & War Injuries PLA, No. 51 Fucheng Road, Beijing, 100048, PR China; b Co-innovation Center of Neuroregeneration, Nantong University Nantong, Jiangsu Province 226007, PR China; c Graduate School of Chinese PLA General Hospital, No. 28 Fuxing Road, Beijing, 100853, PR China; d School of Medicine, Nankai University, Tianjin 300071, PR China

**Keywords:** Spinal cord organoids, Spinal cord extracellular matrix, Neuroepithelium, Neural tube, Motor neuron

## Abstract

Spinal cord organoids (SCOs) are in vitro models that faithfully recapitulate the basic tissue architecture and cell types of the spinal cord and play a crucial role in developmental studies, disease modeling, and drug screening. Physiological cues are required for proliferation and differentiation during SCO culture. However, commonly used basement membrane matrix products, such as Matrigel®, lack tissue-specific biophysical signals. The current study utilizes decellularization process to fabricate tissue-derived hydrogel from porcine spinal cord tissue that retain intrinsic matrix components. This gel system supported an expanded neuroepithelial scale and enhanced ventral recognition patterns during SCO cultivation. Based on the characteristics of the enlarged aggregate size, a technical system for SCO cutting and subculture are proposed to improve the economic feasibility. Finally, the advantage of S-gel in maintaining neurite outgrowth are also found, which suggests its potential application in neural-related microphysiological systems.

## Graphical abstract

**Figure:**
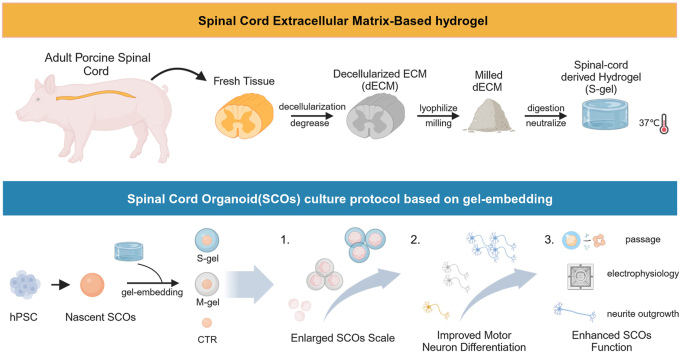

## 1 Introduction

Spinal cord organoids (SCOs) are in vitro models that faithfully simulate developmental events and tissue patterns of the human spinal cord, including the occurrence of the ventral-dorsal and rostral-caudal axis [[1], [2], [3]]. The in vitro development of organoids is not an autonomous process but is influenced by surrounding environmental factors. SCOs typically arise from early embryoid bodies (EBs) exposed to dual SMAD (a drosophila mothers against decapentaplegic protein) signaling inhibition [4], which leads to the formation of neural epithelial structures. These structures, driven by their self-organizing properties and influenced by a combination of exogenous inducing factors, subsequently develop into target tissues. In addition, interactions between epithelial and extracellular matrix (ECM) components play a role in guiding morphogenesis and differentiation during development. The ECM is not only a physical framework but also a determinant factor for tissue specificity. Exposure to a soluble basement membrane matrix (Matrigel) results in rapid polarization and structural rearrangement of neural epithelia, particularly during the establishment phase of apical-basal polarity in neural epithelia [5,6], wrapping early organoids with Matrigel reverses the polarity of neuroepithelial cells on the cell-ECM interface [1,7]. Matrigel serves as an initial source of the ECM that guides the formation of epithelial polarity. However, purified single ECM components such as laminin or type IV collagen cannot achieve this effect, indicating the importance of natural matrices [5].

However, due to the tumor background of Matrigel, such as the high expression of laminin in tumors compared with normal tissues[[8], [9], [10]], it may not fully capture the specific microenvironment of neural tissues. The ECM is produced by different types of cells in the spinal cord, such as neural progenitor cells (NPCs) and glial cells, which play a crucial role in neural development and repair. ECM has been reported to promote the maturation of SCOs based on acellular placental [11] or brain extracellular matrix gels [12] in establishing SCO culture models. However, the spinal cord tissue-specific ECM in developing SCOs has not been extensively studied. Therefore, utilizing SCO models to assess the potential impact of tissue-specific matrices on spinal cord tissue patterning and differentiation is of great significance.

Decellularized spinal cord ECM, a natural biomaterial that retains the biochemical structure and matrix components of native tissue, contains tissue-specific growth factors, peptides, proteins, and other structural biomolecules to support cell attachment, growth, and differentiation [13,14]. Decellularized spinal cord matrix gels, with their unique properties, hold promise for various applications in spinal cord research, such as tissue repair [[13], [14], [15]]. Herein, we generated a temperature-sensitive spinal cord matrix gel system (S-gel) and compared its physicochemical properties with a Matrigel matrix gel (M-gel). Subsequently, both gels were utilized for SCO culture with a suspension culture control (CTR) without exogenous ECM addition. The three cultivation methods were differentiated in terms of epithelial expansion scale during SCO development and the identification of neural precursor cell identity and neuronal recognition. Additionally, we proposed a technical system for subculture of SCO to improve economic feasibility. Finally, our study demonstrated the advantage of S-gel in maintaining neurite outgrowth, which suggests its potential application, such as a 3D substrate for the neuro-involved microphysiological systems.

## 2 Results

### 2.1 Characterization of decellularized spinal cord tissue-derived hydrogels

**Fig. 1:**
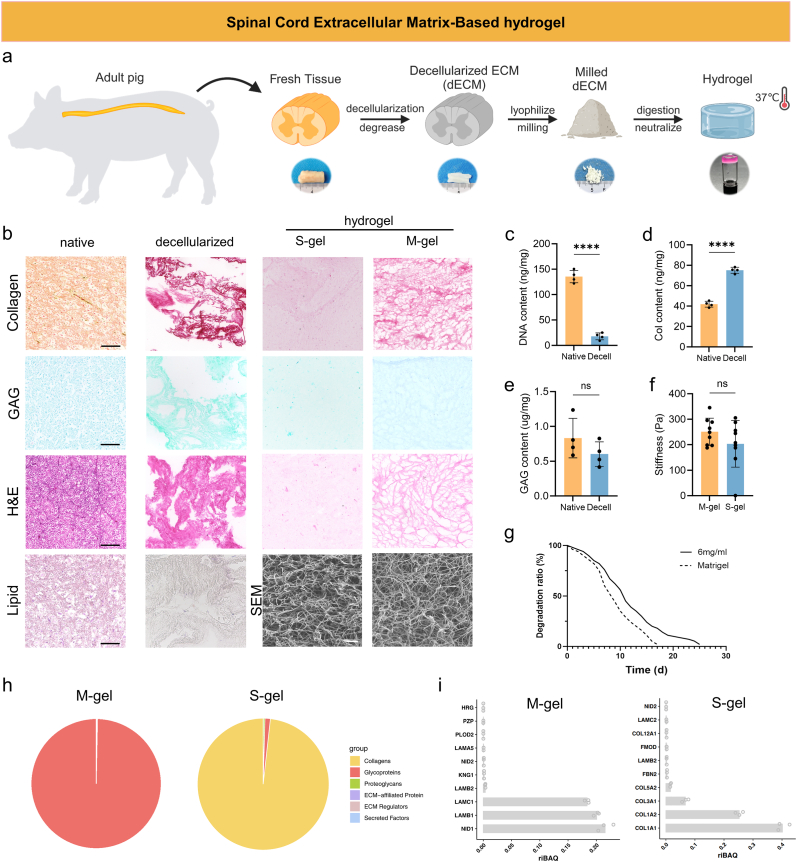
**Preparation and characterization of Spinal Cord tissue-derived ECM hydrogels(S-gel).** a) Schematic illustration of the generation of S-gel derived from decellularized Spinal Cord. b) Protein and lipid content in decellularized spinal cord tissue and gel were assessed using Sirius Red(collagen), Alcian Blue (GAG), H&E, and Oil Red (lipid) staining (Scale bar = 100 μm). And the structure of the gel's protein fiber network was examined through scanning electron microscopy (Scale bar = 2 μm). c) DNA content of spinal cord tissues before and after decellularization [P < 0.0001 (∗∗∗∗)]. d) Collagen content of spinal cord tissues before and after decellularization [P < 0.0001 (∗∗∗∗)]. e) Glycosaminoglycan content of spinal cord tissues before and after decellularization. f) Elastic modulus measured by atomic force microscope (AFM) of S-gel vs. M-gel in 20 μL droplets. g) The degree of degradation of the gels over time. Data are presented as median. h) Composition of total matrisome proteins in M-gel and S-gel, N = 3. i) The most abundant top 10 matrisome proteins in M-gel and S-gel.

Following Good Manufacturing Practice (GMP) principles, the manufacturing process for spinal cord decellularized gel (S-gel) was optimized. This involved aseptically extracting fresh spinal cord tissue, performing decellularization, degreasing, vacuum lyophilization, milling, pepsin digestion, and alkali neutralization (Fig. 1a). Finally, a temperature-responsive hydrogel system was successfully developed, preserving the fundamental tissue morphology and associated ECM components, including collagen and glycosaminoglycans (Fig. 1b–d, e) while effectively eliminating lipids and DNA (Fig. 1b and c). The ECM powder was subjected to pepsin digestion in the presence of hydrochloric acid, followed by neutral pH equilibration and solid gel formation at physiological temperature. Analysis of scanning electron microscopy (SEM) images revealed a highly intricate network of interwoven collagen fibers (Fig. 1b). Rheological properties of different concentrations of S-gel protein and M-gel were assessed using temperature ramp oscillatory rheology (Fig. S1). After exposure to a temperature of 37 °C, both S-gel and M-gel exhibited gel-like characteristics with the storage modulus (G′) surpassing the loss modulus (G″). At approximately 45 °C, the storage moduli of both gels decreased, indicating their melting points. Notably, S-gel at a concentration of 6 mg/mL displayed similar rheological features to M-gel in terms of storage and loss moduli, making it an advantageous choice for cell culture applications. Furthermore, atomic force microscopy (AFM) measurements demonstrated that the elastic modulus of S-gel at a concentration of 6 mg/mL was comparable to that of M-gel (Fig. 1f). However, during in vitro degradation processes, S-gel exhibited a slower degradation rate than M-gel (Fig. 1g), with complete degradation occurring around 17 days for M-gel and 25 days for S-gel.

Proteomic analysis utilizing mass spectrometry (MS) demonstrated that S-gel exhibited a higher abundance of collagen proteins than M-gel, accounting for 98.23 % of the total protein content (Fig. 1h and i), with the most predominant subtypes being collagen, type I, alpha 1 (COL1A1), COL1A2, COL3A1, and COL5A2 in descending order. Glycoproteins constituted approximately 1 % of the overall matrix proteins present in S-gel, with fibrillin 2 (FBN2) and laminin subunit beta 2 (LAMB2) being the most predominant. However, glycoproteins comprised over 99.7 % of the overall matrix proteins present in M-gel, with the most predominant types being nidogen 1 (NID1), LAMA1, LAMB1, and LAMC1.

### 2.2 S-gel affects neuroepithelial (NE) expansion and identification of neural progenitors in nascent SCOs

**Fig. 2:**
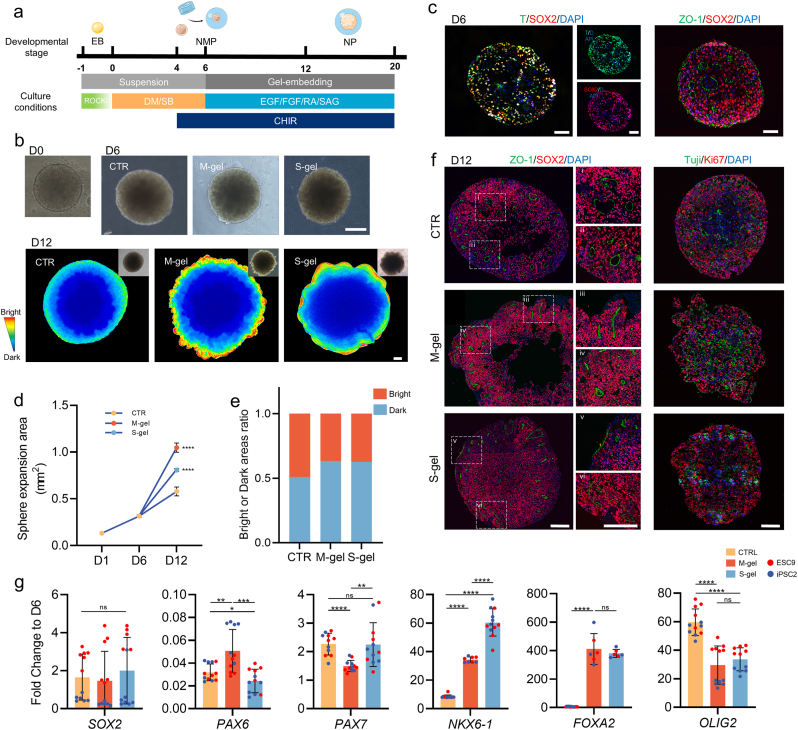
**Gel embedding affects the expansion scale of neural epithelium and the neural progenitor's identity in nascent spinal cord organoids (SCOs).** a) Schematic illustrating the differentiation conditions used to obtain SCOs with gel embedding. EB: Embryoid body; NMP: Neuromesodermal Progenitors; NP: Neural; ROCKi: ROCK inhibitor. b) Brightfield images of SCOs. On the 6th day, SCOs was embedded in Matrigel (M-gel) or spinal cord matrix gel (S-gel), with no gel embedding serving as the control (CTR). Pseudo-color images and bright field images (Upper right) at day 12 were presented. Scale bar = 200 μm. c) Immunofluorescence staining in SCOs cryo-sections at day 6 of in vitro differentiation showing the expression of NMP(T/SOX2) and neuroepithelial cell (NE, ZO-1/SOX2) markers. Scale bar = 50 μm. d) Expansion area of the SCOs sphere at day 1,6, and 12(n = 8 at each time point). Data are presented as the mean ± SD. e) The area ratio of the light and dark field of the SCOs sphere at day 12. The light field and dark field were defined following the established threshold of 16 bit color map of pseudo-color image (n = 6). f) Immunofluorescence staining in SCOs cryo-sections at day 12 of in vitro differentiation showing expression of NE marker. Scale bar = 200 μm. g) Gene expression analysis of neural progenitor domains mitiotic markers in SCOs at day 12 of in vitro differentiation in 4 independent differentiations (n = 3, N = 12). Data are expressed as fold changes relative to that of D6[<0.05 (∗), <0.01 (∗∗), <0.001 (∗∗∗), <0.0001 (∗∗∗∗)].

SCOs were generated starting from embryoid bodies (EBs) formed from Human pluripotent stem cells (hPSCs) (hESC-H9/hiPSC-2), employing the induction protocol for motor neurons. Briefly, NE induction was initiated using the dual-SMAD inhibitor, these aggregates in this phase acquired the identity of neuromesodermal progenitors (NMPs) (Fig. 2a–c). Subsequently, EBs were exposed to fibroblast growth factor (FGF) and Wingless/int1 (WNT) signaling and retinoic acid (RA) signaling for caudal identification of progenitor domains, while the sonic hedgehog (SHH) activator smoothened agonist (SAG) was applied for ventral identification (Fig. 2a). Briefly, spheres were embedded in the gel (M-gel/S-gel) from day 6 onward (Fig. 2b) during the initiation of NE expansion and neural tube (NT) morphogenesis in SCOs (Fig. 2c). These protocols were compared with those without exogenous ECM exposure (CTR) to assess whether ECM supplementation affected nascent SCO development.

A total of 9000 isolated single cells were seeded into each well of an ultra-low-adhesion U-shaped 96-well plate for 24 h to form EBs. EBs were then continuously expanded during the subsequent neural induction phase (Fig. 2d). On day 12, the average planar area of the spheres in a top-down view was 0.5796 mm^2^ in the CTR group, while the average expansion area was 1.047 mm^2^ for the M-gel group and 0.8082 mm^2^ for the S-gel group, indicating that the addition of exogenous matrix gel accelerated organoids expansion (Fig. 2d), with M-gel supporting a faster NE expansion rate than S-gel at this stage (P < 0.0001). For organoid morphology (Fig. 2b), suspended-cultured organoids maintain smooth borders, with the surfaces of the sphere displaying increased translucency, indicative of NE formation. Conversely, organoids exposed to the exogenous gel displayed irregular shapes, suggesting budding at the tissue boundaries (Fig. 2b). The M-gel group exhibited small and dense epithelial buds at the edge of the spheres, while the S-gel group had larger-scale epithelial tissue morphology (Fig. 2b). Meanwhile, the NE scale was described using pseudo-color images with a defined threshold to delineate bright field regions (Fig. 2d). The results showed that gel-encapsulated SCOs exhibited an enlarged NE scale compared with the CTR group (Fig. 2e). Furthermore, the developmental status of the NT was evaluated. Immunofluorescence staining of Zonula occludens-1 (ZO-1), a marker for the apical structure of NT, was performed (Fig. 2f). At day 12, the suspended cultured organoids had already undergone tubular morphogenesis of the NT and exhibited characteristic features, including apical localization of ZO-1 in a tubular pattern, with the radial alignment of sex-determining region Y (SRY)-box 2 (SOX2)-positive nuclei surrounding it (Fig. 2f i-ii). The neural precursor cells in a proliferative state (KI67) displayed a diffuse distribution pattern (Fig. 2f). However, the M-gel group exhibited radially extending NT-like structures at the periphery of SCOs (Fig. 2f ⅲ;-ⅵ), explaining the inherent tooth-shaped budding structure observed at its edges. Notably, at this stage, the internal portion of SCOs enveloped by S-gel exhibited a relatively delayed state of NT development, characterized by both sheet-like neural plate formation (Fig. 2fⅴ) and ongoing folding-based tube morphogenesis (Fig. 2fⅵ). Taken together, these findings suggest that S-gel may influence the overall scale of neuroepithelium through a delayed effect on NT formation.

Subsequently, we explored whether the identity recognition of progenitor domains is affected at this stage. Compared with day 6, the expression level of the neural epithelial marker SOX2 was increased, while the paired box 6 (PAX6) level was reduced (Fig. 2g). At this time, the expression of PAX7, a marker of dorsal progenitor domains, was slightly upregulated in all three groups. Contrarily, the expression level of NK6 homeobox 1 (NKX6-1), a marker of ventral progenitor domains, was significantly increased and higher in the S-gel group than in the M-gel group (P < 0.0001). Furthermore, the expression of motor neuron precursor marker gene oligodendrocyte transcription factor 2 (OILG2) was assessed, and results showed that the CTR group exhibited significantly higher expression than the other two groups, while no significant difference was found between the S-gel and M-gel groups (P = 0.4491). This may be due to a delayed differentiation caused by extensive expansion of NE. Finally, the floor plate marker gene forkhead box protein A2 (FOXA2) was significantly upregulated in both S-gel and M-gel groups, which may be associated with a direct response to ECM contact. The floor plate induced ventral progenitor domains *in vivo* by secreting SHH, which influenced subsequent identity determination.

### 2.3 Extended gel embedding enlarges the neural epithelium of SCOs and affects the rostro-caudal/dorsal-ventral pattern in SCOs

**Fig. 3:**
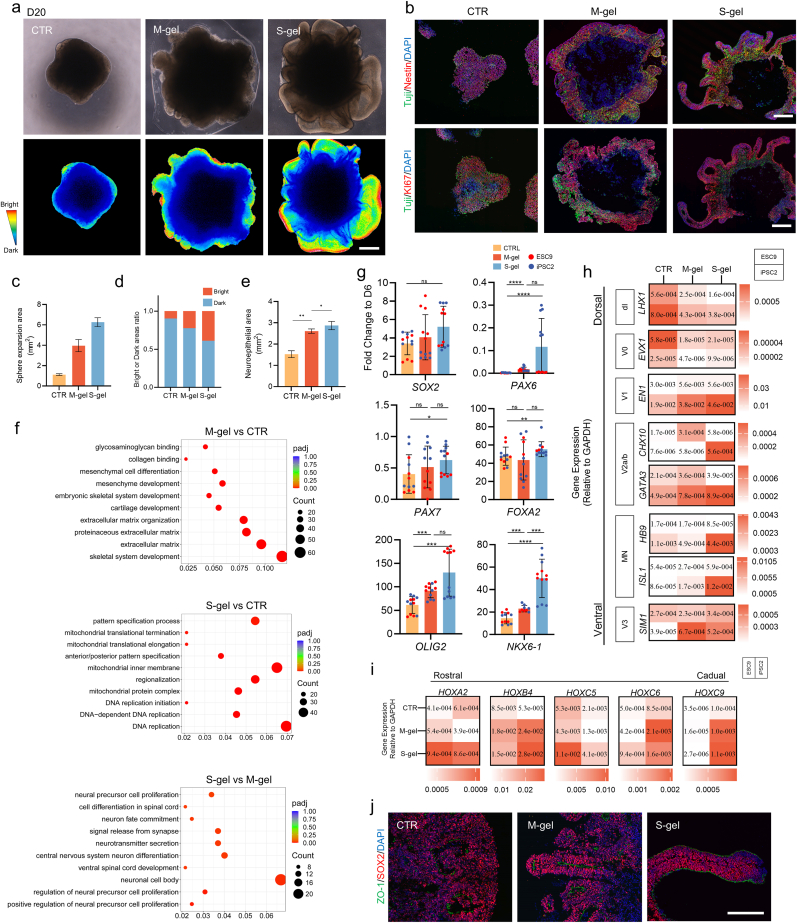
Extended gel embedding enlarges the neural epithelium of SCOs and affects Rostro-Caudal/Dorsal-Ventral pattern in SCOs. a) Bright field and pseudo-color images (Below) of SCOs. Scale bar = 500 μm. b) Immunofluorescence in SCOs cryo-sections at day 20 of differentiation showing expression of neural progenitors (Nestin/Ki-67/Tuji) markers. Scale bar = 500 μm. c) Expansion area of the SCOs sphere at day 20(n = 8). Data are presented as the mean ± SD. d) The area ratio of the light and dark field of the SCOs sphere. Data are presented as mean ± SD. e) Calculate the area of the neuroepithelial region (Nestin^+^) of the SCOs using immunofluorescence staining images of the neuroepithelium(n = 6). Data are presented as the mean ± SD. f) GO enrichment of differential genes (up-regulated) in SCOs transcriptomics at day 20 of differentiation. The mRNA library data were collected from an independent differentiation of the ESC-H9 cell line. g) Gene expression analysis of neural progenitor domains mitiotic markers in SCOs. Data are expressed as fold changes to that of D6[<0.05 (∗), <0.01 (∗∗), <0.001 (∗∗∗), <0.0001 (∗∗∗∗)]. h) Gene expression analysis of genes expressed along the dorso-ventral axes at day 20 of differentiation. Data are presented as the mean. Each cell represents two independent differentiations of a single cell line[Kruskal-Wallis test p = 0.1461(ESC9), p = 0.0285(iPSC2) for *LHX1*, p = 0.6053(ESC9), p = 0.0406(iPSC2) for *EVX1*, p = 0.7571(ESC9), p = 0.0714(iPSC2) for *EN1*, p = 0.0175(ESC9), p = 0.0026(iPSC2) for *CHX10,* p = 0.1506(ESC9), p = 0.1675(iPSC2) for *GATA3*, p = 0.4849(ESC9), p = 0.0463(iPSC2) for *HB9*, P < 0.0001(ESC9), p = 0.0110(iPSC2) for *ISL1*, p = 0.9784(ESC9), p = 0.0500(iPSC2) for *SIM1*]. i) Gene expression analysis of genes expressed along the rostro-caudal axes at day 20 of differentiation. Data are presented as the mean. Each cell represents two independent differentiations of a single cell line[Kruskal-Wallis test p = 0.0068(ESC9), p = 0.0370(iPSC2) for *HOXA2*, p = 0.0024(ESC9), p = 0.0074(iPSC2) for *HOXB4*, p = 0.1187(ESC9), p = 0.1879(iPSC2) for *HOXC5*, p = 0.2393(ESC9), p = 0.6186(iPSC2) for *HOXC6*, p = 0.0072(ESC9), p = 0.9075(iPSC2) for *HOXC9*]. j) Representative Immunofluorescence images of neural tube. Scale bar = 200 μm.

SCOs exhibited a notable expansion in ECM hydrogels with a prolonged culture time. At day 20, the expansion scale was higher in the S-gel group than in the Matrigel-embedded SCOs (Fig. 3a–c). S-gel demonstrated a larger scale of NE characteristics than the CTR or M-gel group (Fig. 3a, b, d, e).

SCOs were analyzed using transcriptomics for a more comprehensive understanding of the developmental aspect of ECOs in gels. Data were obtained from an independent ESC-9 cell line differentiation experiment. Principal component analysis (PCA) revealed that despite variations within groups, the three groups of samples were clearly separated based on the first principal component (Fig. S2). The number of differentially expressed genes (DEGs) is presented in Fig. S3. Furthermore, upregulated or downregulated genes were annotated with Gene Ontology (GO) gene set enrichment to determine the origins of the separation in SCO phenotypes under different matrix conditions (Fig. 3f–S3). The top 10 enrichment results for each comparison were presented. It was found that upregulated mesoderm-mesenchymal developmental gene sets, such as skeletal system development (GO:0001501), cartilage development (GO:0051216), and mesenchyme development (GO:0060485), differed significantly between M-gel and CTR groups. Conversely, downregulated DEGs were annotated with functions related to neuronal development and neurotransmitter signaling regulation (Fig. S3a). For S-gel and CTR groups, upregulated DEGs were mainly related to biological processes (BPs) such as mitochondrial morphogenesis and DNA replication (Fig. 3f). Notably, genes associated with rostro-caudal patterning (HOXB6, SFRP2, HOXD8, HOXC6, HOXD9, HOXB8, GLI2, HOXB7, etc.) were statistically significant, corresponding to the caudalization (brachial-thoracic-lumbar segments) characteristics of the NT. In contrast, downregulated genes were mainly enriched in BPs of later neurodevelopmental stages, such as axonogenesis and neurotransmitter development (Fig. S3b). Intriguingly, a comparison of transcriptional differences between S-gel and M-gel groups revealed that upregulated DEGs were mainly associated with neural progenitor cell proliferation (NESTIN, PAX6, DLL4, OTP, etc.) and ventral spinal cord development (LHX4, OLIG2, ISL1, LHX3, and FOXN4) gene set. Meanwhile, downregulated DEGs were primarily enriched in the mesoderm-mesenchymal developmental gene set, similar to the upregulated gene profile observed in the M-gel versus CTR comparison (Fig. S3c).

To explain the impact of matrix embedding on the rostro-caudal axis pattern of SCOs, we annotated the homeobox (HOX) family genes on the differential gene volcano plot (Fig. S4). At day 20, matrix-embedded organoids exhibited more posterior NT characteristics compared with suspension culture, whereas the S-gel group displayed a more advanced cervical-thoracic developmental profile (HOXA2, HOXC5, HOXB6, and HOXD8) compared to the M-gel group. The findings from genome comparison studies indicate that matrix embedding influences the developmental patterns of SCOs.

Subsequently, real-time PCR (RT-PCR) analysis was conducted on day 20 samples to validate the transcriptomic findings. Similarly, RT-PCR analysis was performed on SCOs for rostral-caudal/dorsal-ventral patterning marker genes (Fig. 3g). The expression level of NE marker PAX6 was higher in the S-gel group than in the M-gel group, especially in the differentiation of the iPSC2 cell line; however, no statistical difference in SOX2 was found between the two groups. The ventral identity of neural progenitor cells was highlighted at this stage due to the addition of exogenous SHH signals. Among them, no significant difference in FOXA2 gene expression was detected among the three groups of samples (Fig. 3g). It is noteworthy that matrix embedding induced a higher ventral identification, especially the identity of motor neurons (OLIG2, ISL1, and HB9), while the S-gel group showed a relatively higher expression level (Fig. 3h). Meanwhile, exposure to gels induced more caudal fates in SCOs (Fig. 3i), while the S-Gel group exhibited increased homology in the cervical (humeral) segments (HOXA2, B4, and C5), in line with transcriptome data. The M-gel group exhibited increased thoracic homology with HOXC6 and C9. Furthermore, SCOs exhibited characteristic NT morphology at this stage, displaying an inward apical polarity. Surprisingly, a total polarity reversal was observed in the S-gel group (Fig. 3j).

### 2.4 S-gel embedding significantly improves motor neuron differentiation efficiency

**Fig. 4:**
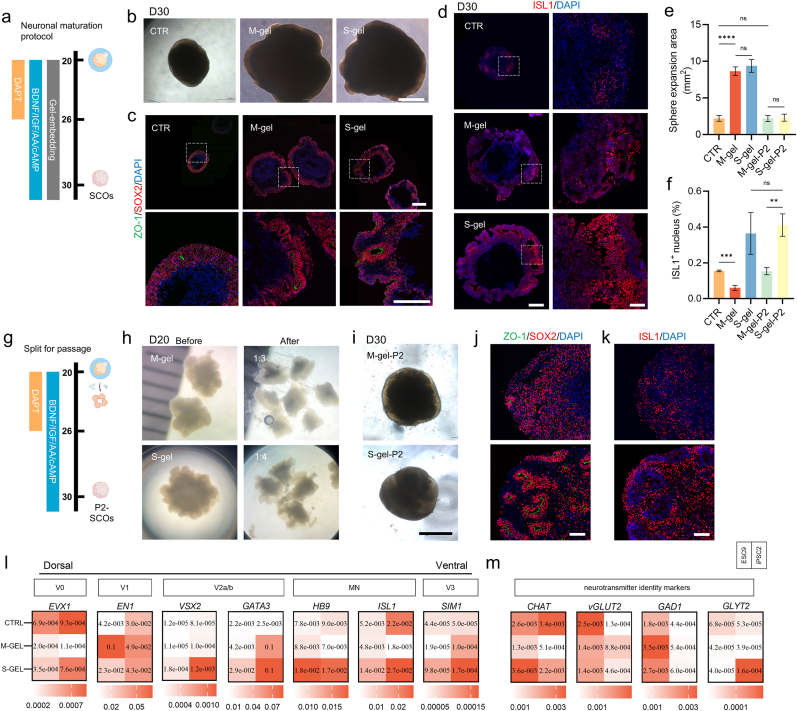
Gel embedding yielded significantly large organoids and enhanced motor neuron differentiation efficiency. a) Schematic illustration of the neuronal maturation protocol for SCOs. BDNF: Brain-derived neurotrophic factor; IGF: Insulin like growth factor; AA: Ascorbic acid; cAMP: Cyclic adenosine monophosphate. b) Bright field images of SCOs at day 30. Scale bar = 1 cm. c) Representative Immunofluorescence images of neural tube. Scale bars, 1 cm (top panel), 500 μm (bottom panel). d) Immunofluorescence in SCOs cryo-sections at day 30 showing expression of motor neuron (ISL-1) marker. Scale bars, 500 μm (left panel), 100 μm (right panel). e) Expansion area of the SCOs sphere at day 30(n = 8). Data are presented as mean ± SD. f) Proportion of ISL-1^+^ nuclei in immunofluorescence staining images. Data are presented as mean ± SD. g) Schematic diagram of the split-and-passage culture protocol for SCOs. h) SCOs were passaged at day 20 by a 1:3 or 1:4 split with syringe needle. i) Bright field images of P2-SCOs at day 30. Scale bar = 1mm. j) Immunofluorescence staining of neural tube of P2-SCOs at D30. Scale bar = 100 μm. k) Immunofluorescence staining of motor neurons (ISL-1) of P2-SCOs at D30. Scale bar = 100 μm. l) Gene expression analysis of neuronal cell type markers in P2-SCOs at day 30 of in vitro differentiation. Data are presented as mean. Each cell represents two independent differentiations of a single cell line[Kruskal-Wallis test p < 0.0001(ESC9), p = 0.0001(iPSC2) for *EVX1,* p < 0.0001(ESC9), p = 0.0129(iPSC2) for *EN1,* p = 0.0004(ESC9), p = 0.0006(iPSC2) for *CHX10,* p < 0.0001(ESC9), p = 0.0006(iPSC2) for *GATA3,* p = 0.0003(ESC9), p = 0.0002(IPSC2) for *HB9,* p = 0.0351(ESC9), p = 0.0709(iPSC2) for *ISL1,* p = 0.3263(ESC9), p = 0.0045(iPSC2) for *SIM1*]. m) Gene expression analysis of neurotransmitter identity markers in P2-SCOs at day 30. Data are presented as mean. Each cell represents two independent differentiations of a single cell line[Kruskal-Wallis test p < 0.0001(ESC9), p < 0.0001(iPSC2) for *CHAT,* p = 0.0343(ESC9), p = 0.0012(iPSC2) for *vGLUT2,* p = 0.0010(ESC9), p = 0.5570(iPSC2) for *GAD1,* p = 0.2110(ESC9), p = 0.0491(iPSC2) for *GLYT2*].

A neuronal maturation induction protocol was implemented on days 20–30 to drive the phenotypic maturation of neurons (Fig. 4a). The gel-embedded SCOs continued to grow at this stage, and no statistical difference in sphere size was found between the two groups (Fig. 4b–e). However, by day 30, the gels had degraded and were no longer visible, and the edges of the exposed SCOs tended to be smooth (Fig. 4b). Although the NT morphogenesis process was completed, the reversed polarity of NT of the S-gel group still existed; however, a folding trend of the epithelium was observed, suggesting the closure of the NT (Fig. 4c). Subsequently, immunofluorescence labeling of the motor neuron marker ISL-1 was performed on frozen sections of SCOs at day 30 to assess differentiation efficiency (Fig. 4d–f). The S-gel group had the highest positive cell rate (P = 0.0356). The differentiation efficiency was lower in the M-gel group than in the CTR group (P = 0.0004).

A larger spheroid volume implies severe internal hypoxia. Caspase-3 staining was conducted to assess the extent of hypoxia-induced apoptotic events (Fig. S5). Gel-embedded spheroids had huge internal cavities at day 30, with the ratio of caspase-3-positive space being higher than that of the CTR group (p < 0.0001). Since these ineffective cavities are insignificant and may even cause negative pressure on neuronal survival, we developed a micromanipulation-based mechanical cutting method using a syringe needle tip to dissect the organoid to exclude dead tissue (Fig. 4h). Experiments were conducted on day 20 of differentiation because by this time the spheres had expanded to a size sufficient for syringe needle tip manipulation and showed large necrotic cavities (Fig. S5). Based on the size of the spheroids, we performed a fixed ratio of passaging on gel-embedded SCOs (M-gel: 1:3, S-gel: 1:4), which we called descendant organoids (P2). The subsequent differentiation protocol was similar to the aforementioned neuronal maturation induction scheme, except for the elimination of the exposure to exogenous ECM (Fig. 4g).

Cutting resulted in an outflow of necrotic tissue within the spheres, and ruptured progeny organoids had reorganized smooth spheres on day 30 (Fig. 4i), while the proportion of internal apoptotic cells was significantly reduced (Fig. S6), at which time the three groups of organoids had relatively uniform sizes (Fig. 4e). Furthermore, the cell viability test results showed that on the 7th or 14th day after the passage operation, the cell viability of P2 - SCOs was higher than that of the unpassaged spheres(Fig. S7).

Similarly, the morphology of NTs in P2 organoids was evaluated on day 30 (Fig. 4j). It was found that the M-gel-P2 group had NTs with sporadic local morphology, while the S-gel-P2 group had a high number of closed NTs. Immunostaining of motor neurons showed that the differentiation efficiency of S-gel-P2 was similar to that of non-passage culture but still higher than that of M-gel-P2, and the differentiation efficiency of M-gel-P2 was higher than that of non-passage culture (Fig. 4f–k). The neuronal identity of a specific set of markers was confirmed on day 30 by RT-PCR, which showed a remarkable ventral neuronal identity of S-gel-P2, including motor neurons and V2a/b interneuron identity (Fig. 4l). The phenotypic traits of neurotransmitter-based identification, with an emphasis on glutamatergic (vesicular glutamate transporter 2 (vGLUT2)) and cholinergic (choline O-acetyltransferase (CHAT)) excitatory neurons, were more evident (Fig. 4m).

**Fig. 5:**
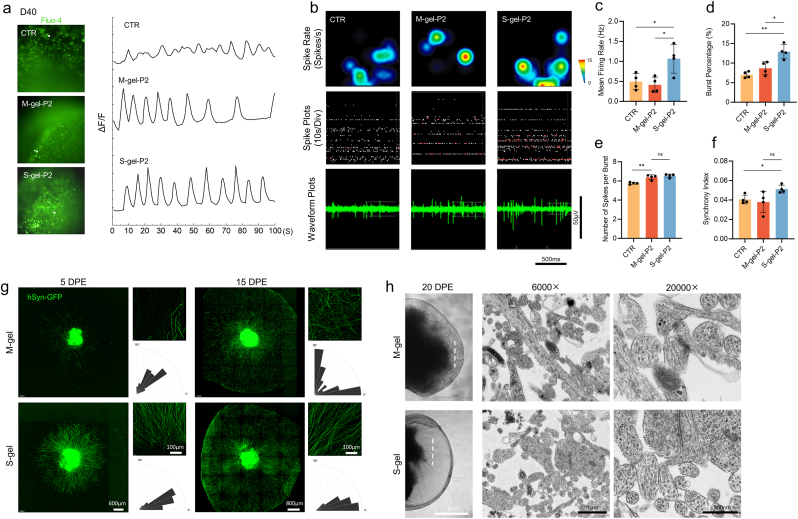
Gel embedding accelerates neuronal maturation and supports robust neurite outgrowth. a) Representative spontaneous calcium responses of SCOs at day 40. DF/F indicates the fluorescence intensity over baseline fluorescence. Arrows show cells analyzed. b) Neural activity patterns in SCOs at day 40. Representative heat maps of electrical activity for each group (top) and electrical oscillation traces of a single electrode (middle), where red dots represent events with more than 5 spike bursts within 100 ms, and electrical signal spikes (bottom) are shown. c) Differences in patterns of neural activity. Bar graphs indicate the mean ± SD of the firing rate[<0.05 (∗), <0.01 (∗∗)]. d) Bar graphs indicate the mean ± SD in mean burst rate. e) Bar graphs represent mean ± SD in number of spikes each burst. f) Synchronization between electrodes. g) Representative images of neurite outgrowth of mature spinal cord organoids (D55) collected at 5 days and 15 days post-embedding and elongation patterns. h) Transmission electron microscope images of axonal sections 20 days after embedding. The white dotted line indicates the location; NF: Neurofilaments; SV: Synaptic vesicles; Mi: Mitochondria.

As a calcium indicator, Fluo-4 was employed to perform spontaneous calcium activity imaging on P2 organoids on day 40 of differentiation. S-gel-P2 and M-gel-P2 exhibited increased response amplitudes compared with the CTR group, suggesting enhanced neuronal maturation (Fig. 5a). Subsequently, multi-electrode arrays (MEAs) were used to record spontaneous neural activity, which refers to the self-generated electrical activity of the neurons. The neuronal excitability level was assessed by analyzing spike-related parameters. Fig. 5b shows a heat map of impulse firing frequency and electrical signal feature descriptions for each group of neurons. S-gel-P2 has a higher average neural firing frequency (Fig. 5c). The occurrence of more than 5 firing events within a unit of time was defined as a burst, indicating the maturation of neuronal advancement (Fig. 5d and e). The S-gel-P2 group had a higher probability of neuronal bursts, with a greater number of neural firings per unit time than the CTR group. Furthermore, the synchronization index was employed to quantify the degree of electrode synchronization for signal acquisition, thereby characterizing neuronal network oscillation. The results revealed no significant difference between the S-gel-P2 and M-gel-P2 groups; however, both groups exhibited higher synchronization than the CTR group (P = 0.0125, Fig. 5f). These findings provide additional evidence supporting enhanced neuronal functional maturity in the S-gel-P2 group.

### 2.5 S-gel provides robust support for axonal growth

Mature S-gel-P2-SCO (D55) cells expressing green fluorescent protein were re-embedded into the two groups of gels to observe neuronal neurite outgrowth (Fig. 5g). It was found that neurite outgrowth was visibly apparent on day 5 post-embedding. However, axons appeared relatively sparse and disordered in the M-gel group but exhibited a strictly radial extension tendency in the S-gel group. On day 15, axons filled the entire gel droplet while maintaining a consistent growth pattern. Transmission electron microscopy (TEM) was conducted on axon cross-sections embedded in the gel for 20 days (Fig. 5h), revealing an enhanced presence of synaptic vesicle structures and mitochondria in the growing axons immersed in S-gel, which explains their robust growth tendency.

## 3 Discussion

Although Matrigel has demonstrated efficacy in maintaining various types of organoids in vitro, including those derived from the nervous system [16,17], it possesses several inherent limitations [18], including the absence of tissue-specific ECM biochemical cues and matrices derived from diseased tissues. Consequently, there is an urgent need for alternative substrates to support organoid culture. The present study developed a decellularized gelatinous matrix sourced from healthy adult pig spinal cord tissue following GMP principles. This raw material preserves crucial biochemical information specific to the tissue microenvironment while also offering wide availability and economic feasibility for potential large-scale commercial applications. Importantly, porcine tissue-derived extracellular framework materials have been extensively utilized for in vitro cultivation of corresponding types of organoids [19], such as gastrointestinal [10,20], hepatic [21], renal [22,23], and cardiac [24] organoids. They have proven advantageous for processes such as stem cell maintenance, differentiation, and vascularization. The current study employed a classical non-ionic detergent-based decellularization process combining Triton-X100 and sodium deoxycholate. The resultant acellular protein matrix obtained through this method exhibited favorable biocompatibility [14,15]. The cell-free matrix obtained from this process effectively preserves essential collagen components and tissue structure while significantly reducing DNA content. Moreover, gel embedding did not induce early apoptosis in SCOs (Fig. S6), indicating reduced residual toxic substances in the gel. Furthermore, a comparison of the mechanical parameters between the S-gel system with a protein concentration of 6 mg/ml and the M-gel system revealed no significant differences between the two systems, except for degradation time. Although S-gel exhibited a considerably slower degradation rate than M-gel, it is noteworthy that the working time of the matrix was 14 days (days 6–20) in our study, significantly diminishing this disparity. Regarding matrix composition, S-gel encompasses intrinsic components such as collagen proteins, laminin proteins, and fibronectin, whereas M-gel includes laminin proteins as intrinsic components and NID as a non-essential component, highlighting its mismatched composition for culturing SCOs.

Flat NE structure serves as the initial stage in human nervous system development, undergoing a series of morphological changes, including induction of polarized NE cells, formation of sheet-like neural plate, and subsequent folding-based tube morphogenesis [1,25], ultimately leading to the development of the central nervous system. The developmental trajectory of spinal cord-like organs summarizes this process. Currently, most region-specific neural organ culture protocols involve suspension culture without matrix contact [2,26]. While these protocols partially capture the principles of neural development, previous studies have demonstrated that early exposure of neural organoids to exogenous Matrigel can induce rapid tissue polarization and NE formation [5], thus necessitating further evaluation. The present study assessed the developmental patterns of two types of gel embedding and suspension culture-derived organoids (CTR). Gel embedding was observed during the early stages of NE development, where the extent of NE generation (days 6–12) was evaluated. Gel exposure influenced the morphology and growth dynamics of early organoid spheres, as evidenced by the centripetal arrangement of ZO-1 and SOX2-positive cells (neural progenitors). Both types of gel embedding supported NE generation at contact surfaces compared with suspension culture. Notably, S-gel facilitated a larger-scale formation of NTs, while M-gel-embedded organoids exhibited advantages in terms of speed and quantity. The use of spinal cord gel during cell analysis seemed to promote the development of cells towards a ventral spinal cord fate, which is the region that gives rise to motor neurons. This ventralization process likely influenced the subsequent identification of specific neuronal types. Moreover, the gel might have facilitated the recognition and function of basement cells, which are important for supporting cell development, by enhancing their interaction with the gel itself. Consequently, the spinal cord gel may have played a role in shaping the final differentiation outcomes of the cells. Overall, genomic comparison on day 20 of induction suggested that M-gel promoted mesodermal tissue differentiation, while S-gel induced ventralization and caudalization features in SCOs.

Intriguingly, the S-gel group exhibited a polarity reversal in the NE morphology on day 20 of differentiation. This phenomenon is rare in conventional organ cultures and only occurs in the presence of a continuous gradient inhibition of transforming growth factor beta signaling during NE formation [27]. In NT structures, the basal components of the NT may be dispensable for further morphogenesis, as this feature is not prominent in some well-formed organ cultures [1]. Therefore, we hypothesize that the inherent protein components of S-gel induce apical condensation in the epithelium. Furthermore, this polarity reversal promptly disappears after gel removal (Fig. 4j).

Importantly, gel-embedded organoids offer a larger expansion area than suspension-cultured SCOs. However, larger organoid volumes may lead to internal cell hypoxia and necrosis due to the generally accepted notion that tissues beyond 100 μm depth experience hypoxia. Therefore, the present study introduces the concept of serial passaging, which involves dividing the parent organoid into descendant organoids (in a ratio of 1:4) while eliminating the internal necrotic tissue. These descendant organoids maintain their spherical morphology and preserve the differentiation characteristics exhibited by parent organoids through self-organization after passaging, effectively remove the existing internal necrotic tissue, and at the same time, can maintain a high level of cell viability. In our perspective, serial passaging not only enhances the cost-effectiveness of SCOs but also ensures their downstream applicability and safety for potential *in vivo* transplantation.

Gel-embedded organoids also demonstrated enhanced ventral neuron identification following phenotypic maturation, thereby preserving the identity of early neural progenitor cells. Moreover, functionally matured organoids exhibited characteristics indicative of excitatory neurons. Further analysis revealed that compared with M-gel, S-gel supported robust axonal extension with significantly higher densities of mitochondria and transport vesicles in the axons. This study lays the groundwork for further exploration of S-gel in various fields, including its potential use within neuro-motor units of organ chips. For such applications, the S-gel's ability to create a 3D environment that promotes extensive axonal connections towards target tissues is particularly crucial.

Unresolved issues remain in this study regarding the timing and mechanism underlying polarity reversal in the internalized neuroepithelia of S-gel. Suitable research tools should be identified to explore how S-gel enhances ventral neuron identification. Additionally, in the scenario of nervous system injury, considering the gradually emerging potential of neural organoids as an alternative therapy [[28], [29], [30]], S - gel has great application potential as a protective carrier for neural sphere transplants. This is because it can support the neurite outgrowth and regulate the local injury - related inflammatory microenvironment. However, in this regard, considering the excellent efficacy of synthetic hydrogels in spinal cord repair[[31], [32], [33]], a comprehensive *in vivo* biological evaluation should be conducted and compared to characterize the specific biological effects of S-gel. What's more, Further optimization of the gel manufacturing process should be considered to minimize the negative effects on nerve regeneration and neuronal protection as much as possible, and a standardized manufacturing process should be established to offset the differences between batches.

## 4 Conclusion

In summary, the present study has developed a technical system that utilizes decellularized spinal cord ECM gel for culturing spinal cord organoids. The gel maintains a larger-scale neural epithelium while facilitating more efficient ventral neuron differentiation. To further enhance the cost-effectiveness of single differentiation protocols, we propose the concept of split passaging. Furthermore, this study validates the feasibility of S-gel as a supporting matrix for nervous related system, opening doors for its application in diverse scenarios.

### 4.1 Experimental section

***Porcine spinal cord decellularization:*** Twelve-month-old Landrace pigs, with an average weight of about 60.0 kg, were purchased from Beijing Huafuyuan Biotechnology Co., Ltd. (Beijing, China). All animal experiments were conducted according to the U.S. National Institutes of Health (NIH) Guide for the Care and Use of Laboratory Animals and were approved by the Experimental Animal Ethics Committee of Beijing Huafuyuan Biotechnology Co., Ltd. (HFYIACUC20230111005). Decellularization was performed as previously described with modifications [15]. To prepare the spinal cord tissue, we first worked under sterile conditions to remove the dura mater from freshly obtained pig spinal cords. The spinal cords were subsequently sectioned into 1 cm cubes and rinsed three times with deionized water to remove any residual blood from the surface. Then, the segments were stirred in aseptic buffer solution in the following order: 3.0 % Triton X-100 (Sigma, #T8787) for 24 h, deionized water for 2 h, 4.0 % sodium deoxycholate (Sigma, #30970) for 24 h, deionized water for 2 h, a mixture of anhydrous ethanol (Macklin, #E809056) and dichloromethane (Macklin, #D807825) (1:2 ratio) for 2 h to remove lipid, and deionized water for 2 h at room temperature and dry conditions. All processes were carried out at 4 °C and stirred on a laboratory rotator at 220 rpm. The cell-free porcine spinal cord tissue was lyophilized in a low-temperature vacuum freeze-dryer (5.5 Pa, temperature −80 °C), ground into an ECM powder using a low-temperature high-speed tissue grinder, and purified by passing through an 80-mesh sieve. The powder was then put in 15 ml aseptic centrifuge tubes, irradiated with cobalt-60, and stored at −80 °C.

***Preparation of spinal cord matrix pre-gel:*** About 10 mg of pepsin (Sigma) at a concentration of 250 μ/mg was mixed in 10 mL of 0.01 M hydrochloric acid (Macklin). The mixture was gently rocked on a shaker for 24 h and filtered through a bacterial filter (Millex-GP). Then, 100 mg of the ECM powder was added to the digested solution and the mixture was continuously shaken on a shaker for 48 h to prepare the digestion solution at a concentration of 10 mg/ml. The pH was adjusted to approximately 7.4 with 1 M sodium hydroxide solution (Macklin, #S835850) at 4 °C. The ionic balance was adjusted using 10 × Dulbecco's Modified Eagle Medium low glucose (DMEM-LG, Servicebio) and deionized water, and a hydrogel solution with concentrations of 6, 8, and 10 mg/ml was prepared. The ECM hydrogel was crosslinked by incubating the solutions at 37 °C for 30 min. All operations were conducted on a laminar flow bench to ensure sterility.

***Analysis of decellularized spinal cord tissue:*** To confirm the effectiveness of the decellularization process, we measured the amount of residual DNA remaining in the spinal cord matrix. Fresh and decellularized spinal cord tissue samples (n = 3) were vacuum-lyophilized for 24 h, weighed, and subjected to a nucleic acid extraction analyzer (QIAcube HT, QIAGEN) for total DNA extraction. The extracted DNA was quantified using a nucleic acid quantitative test kit (#P7589, Invitrogen) following the manufacturer's instructions. The fluorescence intensity was detected at an excitation wavelength of 480 nm and an emission wavelength of 520 nm using Infinite 200 Pro (TECAN). Then, a standard curve was plotted and DNA levels were calculated.

The content of glycosaminoglycan (GAG) in the spinal cord decellularized matrix was determined using 1,9-dimethyl methylene blue (DMMB) colorimetric quantification detection kit (GENMED SCIENTIFIC, GMS19239.2) following the manufacturer's instructions. Approximately 50 μL of the test sample was placed in a 96-well plate and analyzed using a spectrophotometer. The absorbance reading of each sample solution was obtained at a wavelength of 656 nm.

Collagen content in the spinal cord decellularized matrix was measured using the hydroxyproline (HYP) determination kit (Nanjing Jiancheng Biotechnology Research Institute, #A030). To detect the effect of decellularization and lipid removal, the decellularized porcine spinal cord was fixed in 4 % paraformaldehyde, dehydrated with sucrose solution gradient, and cut into 10 μm sections, which were then subjected to hematoxylin and eosin staining (Solarbio, #G1120) and oil red O fat staining.

***Proteomic assay:*** Proteomic data collection and analyses were performed by Genedenovo Biotechnology Co., Ltd. (Guangzhou, China). After extraction (n = 3), proteins were quantified using the bicinchoninic acid assay. Sodium dodecyl-sulfate polyacrylamide gel electrophoresis (SDS-PAGE) band types for each group of proteins were detected. Subsequently, sequential proteolytic digestion was performed, followed by peptide quantification. The mixture of peptides was separated using a special technique called high-pH reversed-phase separation. Then, the separated peptides were analyzed by a powerful instrument called an Orbitrap Fusion Lumos mass spectrometer. This instrument can automatically switch between two types of analysis: a basic overview (MS) and a more detailed analysis (MS/MS) of each peptide. Raw data were analyzed using Spectronaut X (Biognosys AG) in a consolidated search library, and nano-liquid chromatography-tandem mass spectrometry (nano-HPLC-MS/MS) data were acquired using the UniProt database. Proteins were quantified using the iBAQ algorithm integrated within the MaxQuant platform, which calculates iBAQ values by dividing the observed ion intensity by the theoretical number of peptides that can be produced from each protein, thus providing an accurate reflection of relative protein molarity. Using the MatrisomeDB database (http://matrisomeproject.mit.edu), a specialized repository for extracellular matrix (ECM) proteomics, a detailed categorization of identified ECM proteins was performed. These proteins were then classified into six major categories: collagens, glycoproteins, proteoglycans, ECM-affiliated proteins, ECM regulators, and secreted factors, to enrich our understanding of ECM protein composition and function.

***Rheological determination:*** During measurement, the initial temperature of the rotational rheometer (Anton Paar, #MCR 302) was set to 0 °C, and the final measurement temperature was adjusted to approximately 40.2 °C, for a total duration of 608s. The specific parameters measured included an oscillatory strain frequency at a rate of 6.8 rad/s and shear strain at a level of 42 %. A series of concentrations of S-Gel and Matrigel were prepared and tested at a temperature of −4 °C. Subsequently, viscoelastic parameters of water-based gels were measured under different temperatures successively.

***Scanning electron microscopy:*** To prepare S-Gel and M-Gel samples for electron microscopy, we first fixed them in a specialized solution and stored them at 4 °C. Following a wash with PBS, the samples were further fixed in a solution containing 2 % osmic acid. Following additional PBS washes, they were dehydrated through a graded series of ethanol and isoamyl acetate solutions. Finally, the samples were freeze-dried using a vacuum lyophilizer for 24 h. Mounted onto stubs and sputter-coated with gold, the samples were then ready for observation and imaging of their internal structures using a SEM in cross-section.

***AFM measurements:*** The AFM force spectroscopy analyses were conducted using a AFM BioScope Resolve (Bruker) in a fluid at 24 °C (n = 3). A colloidal probe (Nano And More, CP-PNPL-SiO-E−5, 0.08 N/m) with a glass sphere of 15 nm diameter was utilized and calibrated through thermal tuning on a glass substrate before each measurement. The force curves were acquired under a force volume mode at a scan size of 1 μm, ramp rate of 1 Hz, ramp size of 15 μm, and a deflection error trigger threshold of 100 nm. To determine the apparent Young's modulus using a fixed Poisson's ratio of 0.5, the force curves were analyzed with the Hertz model within in the Nanoscope Analysis software (Bruker).

***In vitro degradation assay:*** The cross-linked S-gel samples and M-Gel were soaked in PBS at 37 °C. The samples were dried at 50 °C under vacuum to remove moisture, and their weights were measured at predetermined time points. The degradation rate was calculated using the formula: degradation rate = (current weight of hydrogel/initial weight of hydrogel) × 100 %.

***hESC culture:*** Human embryonic stem cells (hESCs) were used in line with the local ethical regulations. The stem cell line exhibited a normal karyotype as determined by genomic stability testing. Human ESC-H9 (DC-02) and iPSC-2 (RC01001-B, female, 0 years old, umbilical cord-derived) were cultured in a 6-well plate coated with hESC-Qualified Matrigel (Corning, #354277), without feeders, at 37 °C, 5 % CO_2_ environment using ncTarget hPSC Medium (Shownin Biotechnologies Co., Ltd, #RP01020). The medium was changed daily and cells were passaged when they reached a confluency of 90 % using the hPSC Dissociation Reagent (Shownin Biotechnologies Co., Ltd, #RP01007). Mycoplasma testing was performed monthly on the culture plate. To minimize potential variations due to prolonged culturing, the hESCs used throughout this study were all below passage 45.

***Generation of SCOs from hESC:*** The SCOs were prepared from hESCs as previously described [2] with some modifications. Briefly, on day −1, confluent hESCs were detached from Matrigel-coated plate using Gentle Cell Dissociation Reagent (Gibco, #A11105-01) and further mechanically dissociated into single cells. Cells were counted and resuspended in the hPSC Medium containing 10 μM Y-27632(MCE, #HY-10071) at a density of 9000 cells per 100 μl in the medium. Next, 9000 cells were added to each well of a 96-well ultra-low-attachment U-bottom plate (Corning, #7007). After 24 h, on day 0, when embryoid bodies had formed, 75 μl of hPSC Medium was removed, and 100 μl of KSR medium containing of DMEM/F12 (Life Technologies, #11330–032), 20 % knockout serum replacement (Life Technologies, #10828028), 1X non-essential amino acids (Gibco, #11140–035), 1X GlutaMax (Gibco, #35050038), 1X β-mercaptoethanol (Gibco, #21985023), and supplemented with 5 μM dorsomorphin (Sigma, #P5499) and 10 μM SB-431542 (Sigma-Aldrich, #S4317), was added to each well. This medium was replaced daily within the first six days. From day 4 to day 20, the WNT activator CHIR-99021(3 mM; Sigma, #SML1046) was utilized for caudalization.

On day 6, spheroids were either embedded in the gel or left unembedded (CTR) and transferred to a neural induction medium containing Neurobasal-A (Life Technologies, #10888022), 1X N-2 supplement (Life Technologies, #17502048), 1X B-27 supplement without vitamin A (Life Technologies, #12587010), 1X GlutaMax (Gibco, #35050038), and supplemented with RA (0.1 μM; Sigma-Aldrich, #R2625), EGF (20 ng/ml; R&D Systems, #236-EG), FGF-2 (10 ng/ml; R&D Systems, #233-FB-025), and the SHH modulator smoothened agonist (SAG, 1 mM; Millipore, #566660) until day 20, with medium changes every day.

From day 20 onward, organoids are subjected to cutting procedures or directly transferred to the neural induction medium supplemented with BDNF (20 ng/ml, Peprotech, #450–02), IGF-1 (10 ng/ml; Peprotech, #100–11), L-Ascorbic Acid (AA, 200 nM; Wako, #321–44823) and cAMP (50 nM; Sigma, #D0627). The Notch pathway inhibitor DAPT (2.5 mM; STEMCELL technologies, #72082) was added for neuralization from day 20 to day 26. Medium was changed every three days. Fig. 2, Fig. 4a, g displays the schematic illustration of the SCOs recipe. Specifically, for gel embedding, the Organoid Embedding Sheet (STEMCELL technologies, #08579) was placed into a sterile 100 mm dish. Using a 200 μL pipette tip, the organoids were drawn with 25–50 μL of medium from plate and transfer to the embedding surface. Excess culture medium was then gently removed from each organoid using a 200 μL pipette tip. Next, 15 μL drops of cold Matrigel/Spinal cord matrix gel were added dropwise onto each organoid. The organoids were then carefully repositioned to the center of the gel droplets using the pipette tip. Finally, the dish containing the organoids was placed in a 37 °C incubator for 30 min to allow the gel polymerization. Finally, using neural induction medium, they were gently washed off the sheet and into the well of a 6-well ultra-low adherent plate (Corning, #3473), each well accommodated up to 12 droplets, with 3 ml of medium. Neurite outgrowth assessment was performed using the same procedure.

***Passage procedure:*** For the cutting operation, the gel-embedded spheroids were transferred to a 100 mm sterile dish and processed under a stereomicroscope in a sterile environment. Two syringe tips (29G′) were used: one to gently remove the solidified gel and pierce the center of the spheroid, and the other to perform a gentle cut, taking care to avoid damaging the neural epithelial part on the surface of the spheroid. The culture medium was then gently blown on to facilitate the expulsion of necrotic tissue from the fragments. After washing the fragments three times with fresh medium, they were transferred to an ultra-low adherent plate for continued cultivation.

***cell viability assay:*** Carefully collect the SCOs spheres from the culture plates on day 27, and day 34, ensuring that spheres from each time point are collected separately. Gently transfer the spheres from each time point into a sterile 96-well plate, placing 5 spheres per group to ensure experimental reproducibility. Add a mixture of alamarBlue™ cell viability assay reagent (A50101, Invitrogen) and complete culture medium to each well containing spheres, in a ratio of 1:9. Incubate the samples at 37 °C for 1 h. Take 200ul of the supernatant and measure the fluorescence intensity using a fluorescence spectrophotometer at an excitation wavelength of 560 nm and an emission wavelength of 590 nm.

Take the samples that have completed the alamarBlue™ cell viability assay and gently transfer them into Eppendorf (EP) tubes, being careful to avoid breaking or deforming the spheres. Prepare the necessary reagents and buffers according to the instructions of the DNA extraction kit (51304, QIAGEN). Add an appropriate amount of cell lysis buffer to the EP tubes to ensure full contact between the spheres and the lysis buffer, promoting cell lysis. After lysis, follow the manufacturer's instructions and use the nucleic acid quantitation kit (P7589, Invitrogen) to quantify the extracted DNA. Measure the fluorescence intensity using the Infinite 200 Pro (TECAN) at an excitation wavelength of 480 nm and an emission wavelength of 520 nm. Then, plot a standard curve and calculate the DNA content.

***Real-time PCR:*** For RT-PCR analysis, 2–3 spheroids were pooled per sample. mRNA was extracted using the RNeasy Plus Micro kit (QIAGEN, #74034), and cDNA was synthesized by reverse transcription with the HiScript III All-in-one RT SuperMix Perfect for qPCR (Vazyme, #R333-01). RT-PCR was then performed using the Taq Pro Universal SYBR qPCR Master Mix (Vazyme, #Q712-02) on a QuantStudio 3 system (Applied Biosystems). The primers used are listed in Table 1.

**Table 1:** List of genes and sequences of primers used for RT-PCR.

**Gene**	**Forward primer**	**Reverse primer**
*GAPDH*	CATGAGAAGTATGACAACAGCCT	AGTCCTTCCACGATACCAAAGT
*SOX2*	CAAGCTCCTTCAACTGGTTCTGT	CTTAGAATGATGCAAGCCAGGTC
*PAX6*	TCTTTGCTTGGGAAATCCG	CTGCCCGTTCAACATCCTTAG
*HOXA2*	CCCCTGTCGCTGATACATTTC	TGGTCTGCTCAAAAGGAGGAG
*HOXB4*	TCCTCGTTTTCAGCTTTGGC	TCATTTGTTAGCGGGTGTCG
*HOXC5*	AGAGCCCCAATATCCCTGC	CGGTGGGAAAGTGATGCTT
*HOXC6*	ACAGACCTCAATCGCTCAGGA	AGGGGTAAATCTGGATACTGGC
*HOXC9*	ACTCGCTCATCTCTCACGACA	GACGGAAAATCGCTACAGTCC
*PAX7*	AAAACCCAGGCATGTTCAGC	ACTAAACCTGAGGGCACAGTG
*OLIG2*	GGACAAGCTAGGAGGCAGTG	ATGGCGATGTTGAGGTCGTG
*NKX6-1*	ACGAGACCCACTTTTTCC	CCCAACGAATAGGCCAAACG
*FOXA2*	GCGACCCCAAGACCTACAG	GGTTCTGCCGGTAGAAGGG
*EVX1*	AACCCCAATGCAAGCTTCAC	TGGGCTTCAAAGGAAAACCC
*EN1*	CCAGTTTCGTTTTCGTTGCC	ACACACTCTCGCACACAAAG
*CHX10*	TGCAACGCTCACTGGTTTTG	TGGCAGGCAATTCACAATGC
*GATA3*	GCCCCTCATTAAGCCCAAG	TTGTGGTGGTCTGACAGTTCG
*HB9*	AGCACCAGTTCAAGCTCAAC	TGGCCTTTTTGCTGCGTTTC
*ISL1*	GCGGAGTGTAATCAGTATTTGGA	GCATTTGATCCCGTACAACCT
*SIM1*	CGCGGACTAGGAGGGAGAA	GTCGTGAGTCTGATTATGGATGC
*CHAT*	ACATGATTGAGCGCTGCATC	ACTTGTCGTACCAGCGATTG
*VGLUT2*	GGGAGACAATCGAGCTGACG	TGCAGCGGATACCGAAGGA
*GAD1*	ATGCAACCAGATGTGTGCAG	TGCCCTTTGCTTTCCACATC
*GLYT2*	CAACGCGCTGCACTGTAAG	CAGGGGTATTGTTCCGCTCC
*FABP7*	GGCTTTGCCACTAGGCAGG	TGACCACTTTGTCTCCTTCTTGA
*SOX10*	CCTCACAGATCGCCTACACC	CATATAGGAGAAGGCCGAGTAGA

***Transcriptomic assay:*** Three biological replicates were set up for each group, and each sample had 5-7 spheroids. Spheroids were lysed with TRIzol (Life Technologies, #15596018) for 30 min at room temperature, and then snap-frozen in liquid nitrogen, stored at −80 °C, and transported on dry ice. The library construction, data collection, and analyses were performed by Novogene Co. Ltd. (Beijing, China). After mRNA extraction, total mRNA amount and integrity were measured using an Agilent 2100 Bioanalyzer. After RNA extraction from the samples, we built sequencing libraries and performed quality checks to ensure they met sequencing standards. Samples that passed these checks were sequenced using the Illumina NovaSeq 6000 platform. Gene expression levels were then calculated as Fragments Per Kilobase of transcript per Million mapped reads (FPKM). To account for variations and ensure a normal distribution, we log-transformed the FPKM values. Next, we used the DESeq2 software (version 1.20.0) to analyze the gene expression data and identify genes whose expression levels differed significantly between the two experimental conditions. Genes with a adjusted p-value (padj) ≦ 0.05 and an absolute log2 fold change ≧ 1 were considered differentially expressed. Finally, we used the Cluster Profiler (version 3.8.1) software to perform GO and KEGG pathway enrichment analysis on these differentially expressed genes. This will allow us understand the biological processes and pathways potentially affected by the experimental conditions.

***Cryopreservation and immunohistochemistry:*** Briefly, spheroids were fixed in 4 % paraformaldehyde (PFA in PBS, Electron Microscopy Sciences) for 12 h at room temperature. They were then washed three time with PBS, and subjected to sucrose cryopreservation (30 % sucrose in PBS for 24–48 h or until samples sank to the bottom), embedded at a ratio of 1:1, 30 % sucrose: FSC (Surgipath FSC 22 Blue, Leica, #3801481) and freezing.

To conduct immunocytochemistry, 16 mm thick sections were cut using a cryostat (Leica). and the sections were washed with PBS to remove excess OCT, blocked for 1 h at room temperature (10 % normal goat serum (NGS), 0.3 % Triton X-100 diluted in PBS), and incubated overnight at 4 °C with primary antibodies in blocking solution. Next day, the cryosections were washed with PBS and then incubated with secondary antibodies for 2 h at room temperature. Alexa Fluor secondary antibodies (Life Technologies) were diluted in blocking solution at 1:1000. They were washed with PBS and the nuclei were stained with DAPI. The primary antibodies used in this experiments are listed in Table 2.

**Table 2:** List of antibodies used for immunocytochemistry.

Antibody	Species	Dilution	Company (Catalogue number)
SOX2	Rabbit	1:1000	Abcam (ab97959)
Brachyury(T)	Mouse	1:100	Thermo Fisher (14-9770-82)
Ki67	Rabbit	1:250	Abcam (ab16667)
ZO-1	Mouse	1:100	Thermo Fisher (33–9100)
Nestin	Rabbit	1:100	Abcam (ab105389)
beta III Tubulin(Tuji)	Mouse	1:1000	Abcam (ab78078)
ISL-1	Rabbit	1:100	Thermo Fisher (PA5-27789)
Cleaved Casepase-3	Rabbit	1:100	Abcam (ab2302)

***Calcium imaging:*** The calcium flow of the organoids was monitored by incubating them with a calcium chemical indicator Fluo-4 AM (3uM, Life Technologies, #C34852) for 30 min at 37 °C. Subsequently, the organoids were washed three times with DPBS. Calcium images were captured at 1 frame per second for 100 s at 37 °C using a high-content analysis system (PerkinElmer, Operetta CLS). Changes in fluorescence intensity were analyzed using ImageJ. The ΔF/F trajectory for each region of interest (ROI) was calculated by dividing the fluorescence over time by the baseline fluorescence. Signal decay was controlled by subtracting the mean baseline fluorescence. Signal attenuation was calculated by subtracting the average fluorescence of the background. ΔF/F = (F_ROI_-F_b_)/F_b_, where F_ROI_ and F_b_ represent the average fluorescence value of ROI and whole frame, respectively.

***Viral labeling of SCOs:*** To label neural spheroids, we diluted 0.5 μl of rAAV-hSyn-EGFP-WPRE-hGH-pA virus solution (Brainvta) in 200 μl (at a titer of 1.0 × 10^11^ vg/ml) of neural induction medium and added the mixed medium to a 1.5-ml EP tube. Three spheroids (S-gel-P2-SCOs) were then transferred into the medium and viruses tube. The tube was placed in an incubator overnight at 37 °C, 5 % CO_2_. The next day, extra 800 μl of fresh neural induction medium was added to the tube and further incubated for 24 h. On day 3, the spheroids were washed with 1 ml of fresh neural induction medium and then transferred back to their culture plate. Expression of the GFP was usually observed 14 days after AAV infection.

***Multi-electrode array (MEA) recording:*** P2-SCOs at D40 were seeded on 6-well MEA plates (Axion Biosystems). The cells were allowed to achieve electrical stability. Subsequently, the recordings were conducted in a Maestro MEA system using the AxIS Software Spontaneous Neural Configuration (Axion Biosystems). Using Axion Biosystems’ Neural Metrics Tool, active electrodes were set to more than 5 spikes/min. Bursts/electrode were defined as inter-spike interval (ISI) threshold requiring minimally 5 spikes with a maximum ISI of 100 ms. Network bursts required at least 15 spikes under the same ISI. The synchrony index utilized a cross-correlogram window of 20 ms.

***Transmission electron microscopy:*** The gel droplets containing organoid and axon were fixed with fixative for TEM (Servicebio, #G1102) for 15 min at room temperature, and placed into a petri dish containing fixative, and the axon is transversely cut using a scalpel. Tissue blocks were transferred into an EP tube using a fresh fixative. Next, samples were then moved to 4 °C for processing. The fixative was removed and replaced with 1 % OsO_4_ (Ted Pella Inc, #18456) and samples were then allowed to rotate to room temperature for 2 h. After the 3 times rinse, samples were stained in1% uranyl acetate for 2 h while rotating. Samples were then dehydrated in a series of ethanol washes for 20 min each at room temperature: 50 % ethanol, 70 % ethanol (at 4 °C overnight), 95 % ethanol (while allowing to warm at room temperature) and, lastly 100 % ethanol. Finally, two changes of acetone were made for 15 min. Resin penetration and embedding as followed: Acetone (Sinaopharm, #10000418): EMBed 812(SPI, #90529-77-4) = 1:1 for 2–4 h at 37 °C; Acetone: EMBed 812 = 1:2 overnight at 37 °C; pure EMBed 812 for 5–8 h at 37 °C; Pour the pure EMBed 812 into the embedding models and insert the tissues into the pure EMBed 812, and then keep in 37 °C oven overnight. The embedding models with resin and samples were then placed in a 60 °C oven for polymerization for over 48 h. The resin blocks were sectioned into 1.5 μm slices using a semi-thin microtome, stained with toluidine blue, and positioned under a light microscope for examination. Subsequently, the resin blocks were cut to 60–80 nm thin on the ultramicrotome, and the tissues were collected onto the 150 meshes cuprum grids with formvar film. 2 % uranium acetate saturated alcohol solution in darkness for 8 min, rinsed in 70 % ethanol for 3 times and then rinsed in ultra-pure water for 3 times. To prepare the samples for TEM, the samples were stained with a 2.6 % lead citrate solution for 8 min to prevent CO2 staining. Following the staining, the samples were thoroughly rinsed three times with ultrapure water to remove excess stain. After rinsing, the samples were carefully dried using filter paper. The dried samples were then placed on grids and allowed to dry overnight at room temperature. Finally, the prepared samples were examined using a Hitachi HT7800 microscope, and images were captured using the Hitachi TEM system.

***Imaging and analysis:*** Brightfield images of organoids were recorded at different time points (days 0, 6, 12, 20, 30) using an inverted phase contrast microscope (Olympus). Stained slides were imaged with a confocal panoramic scanner (Pannoramic, 3DHISTECH). Organoid morphology was quantified at different timepoints using ImageJ software. Brightfield images were analyzed to measure whole organoid area. The polygon selection tool was used to meticulously trace the outer boundaries of each organoid, and the enclosed area was automatically calculated by ImageJ.

To quantify ISL1-positive cells on the entire organoids, up to 12 randomized areas were selected within the germinal zone of each organoid and nuclei that were positive for ISL1 were manually counted. The numbers of ISL1^+^ cells were normalized based on the DAPI staining.

***Statistical analyses:*** Statistical analyses were conducted using an unpaired Student's t-test to compare two groups. One-way ANOVA with Kruskal-Wallis test was employed to compare multiple groups. All analyses were performed using GraphPad Prism 9 software, and the data were presented as the mean or mean ± SD. Statistical significance was set at *P < 0.05*.

## CRediT authorship contribution statement

**Yanjun Guan:** Writing – review & editing, Writing – original draft, Software, Methodology, Formal analysis, Data curation. **Zhibo Jia:** Visualization, Methodology, Formal analysis, Data curation. **Xing Xiong:** Software, Methodology, Formal analysis, Data curation. **Ruichao He:** Visualization, Software, Methodology, Formal analysis, Data curation. **Yiben Ouyang:** Visualization, Data curation. **Haolin Liu:** Formal analysis, Data curation. **Lijing Liang:** Formal analysis, Data curation. **Xiaoran Meng:** Formal analysis, Data curation. **Ranran Zhang:** Formal analysis, Data curation. **Congcong Guan:** Software, Methodology. **Sice Wang:** Software, Formal analysis. **Dongdong Li:** Visualization, Software. **Yuhui Cui:** Data curation. **Jun Bai:** Visualization. **Jinjuan Zhao:** Data curation. **Haoye Meng:** Visualization, Software. **Jiang Peng:** Writing – review & editing, Writing – original draft, Validation, Supervision, Resources, Investigation, Funding acquisition. **Yu Wang:** Writing – review & editing, Writing – original draft, Supervision, Resources, Project administration, Investigation, Funding acquisition.

## Funding

This study was funded by the National Natural Science Foundation of China (32171356) and the Beijing Natural Science Foundation (L222147).

## Declaration of competing interest

The authors have no conflict of interest to declare.

## Data Availability

Data will be made available on request.
