# Supplementary material for: Tissue-specific extracellular matrix for the larger-scaled expansion of spinal cord organoids
Source: Mater Today Bio. 2025 Feb 11;31:101561. doi: 10.1016/j.mtbio.2025.101561 (PMC11904521; doi:10.1016/j.mtbio.2025.101561)
Supplement: Multimedia component 1 [file mmc1.docx]

Supporting Information

**Tissue-specific** **extracellular matrix for the large-scaled expansion of spinal cord organoids**

Authors: Yanjun Guan,^1,2,3^† Zhibo Jia,^1^† Xing Xiong,^1,3^† Ruichao He,^1,4^† Yiben Ouyang,^1,4^ Haolin Liu,^1,3^ Lijing Liang,^1,3^ Xiaoran Meng,^1^ Ranran Zhang,^1^ Congcong Guan,^1^ Sice Wang,^1,3^ Dongdong Li,^1^Yuhui Cui,^1,3^ Jun Bai,^1^ Jinjuan Zhao,^1^ Haoye Meng,^1^ Jiang Peng,^1,2^* Yu Wang,^1,2^*

**
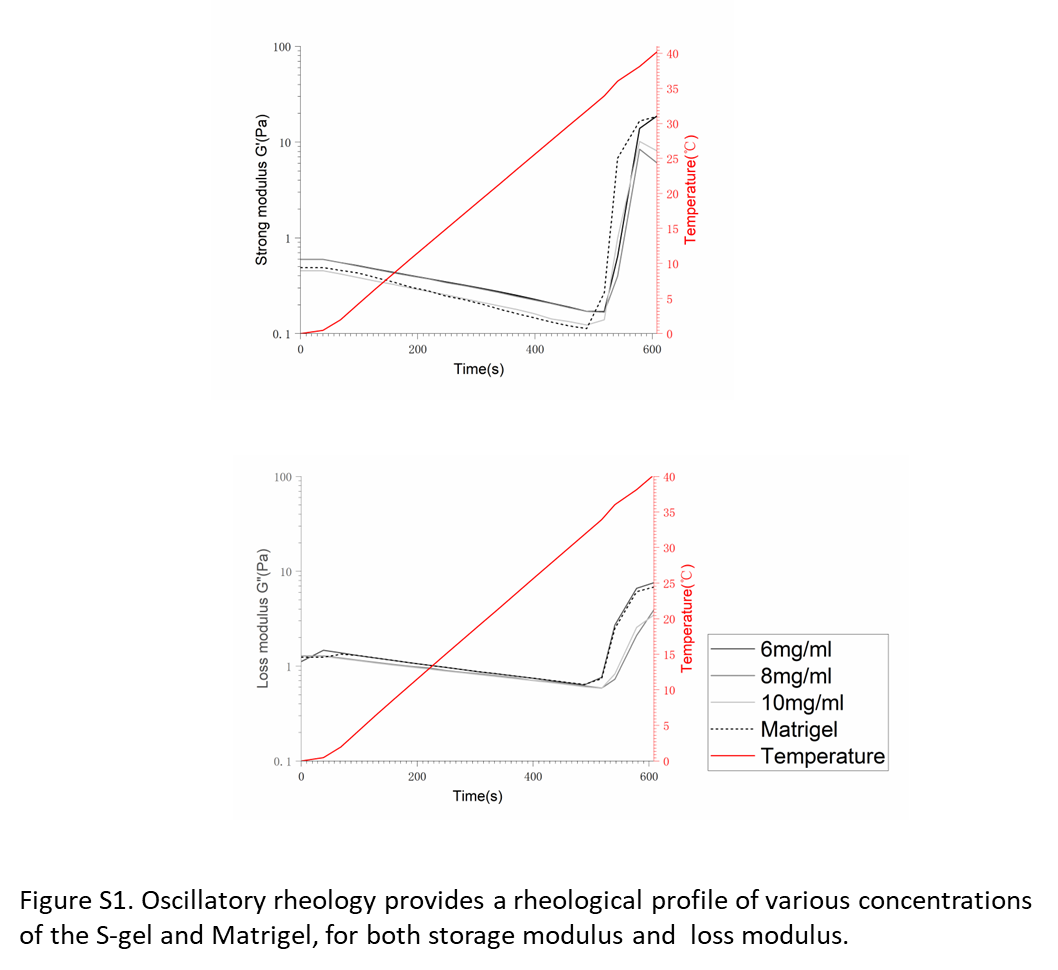
**

**
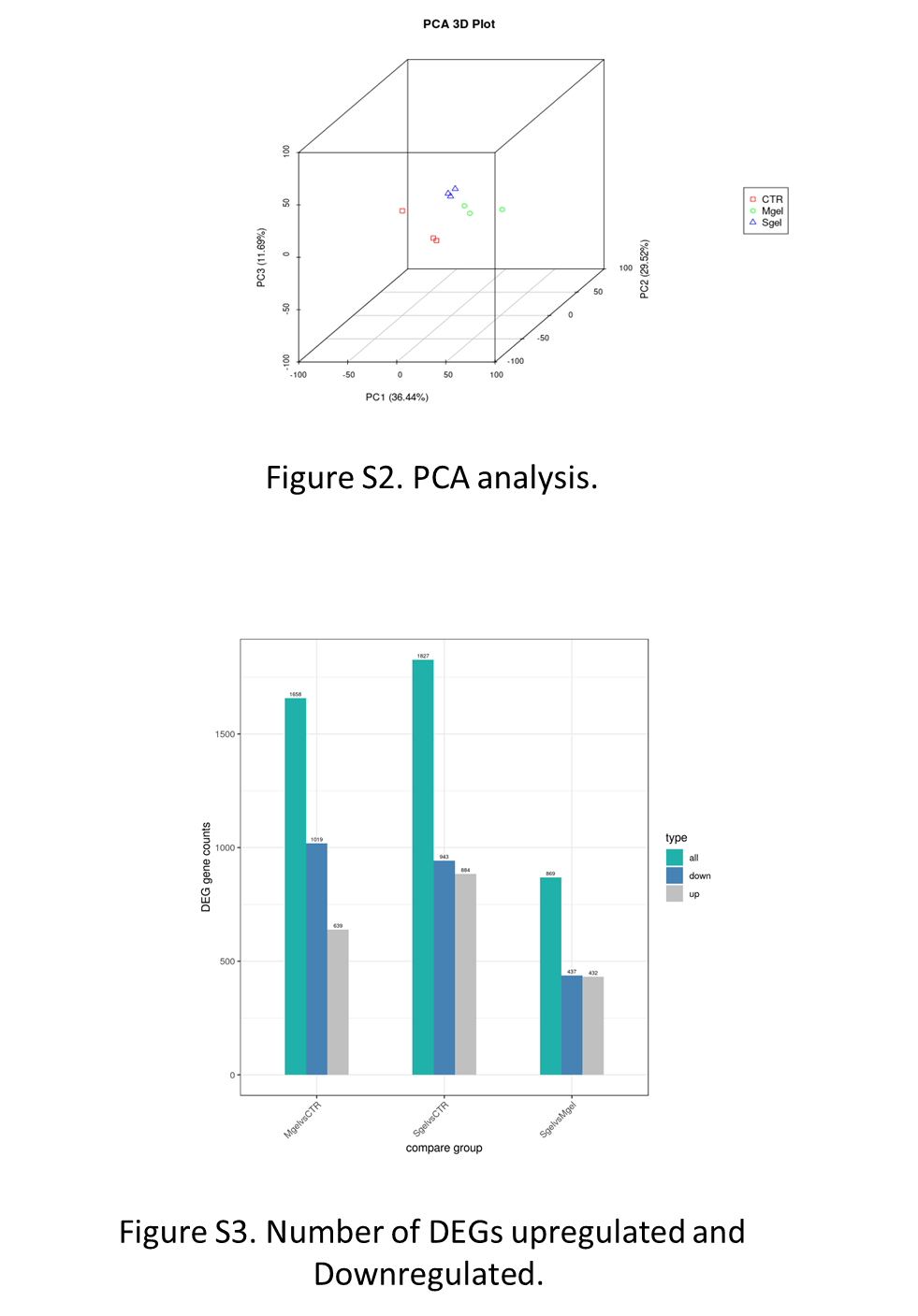
**

**
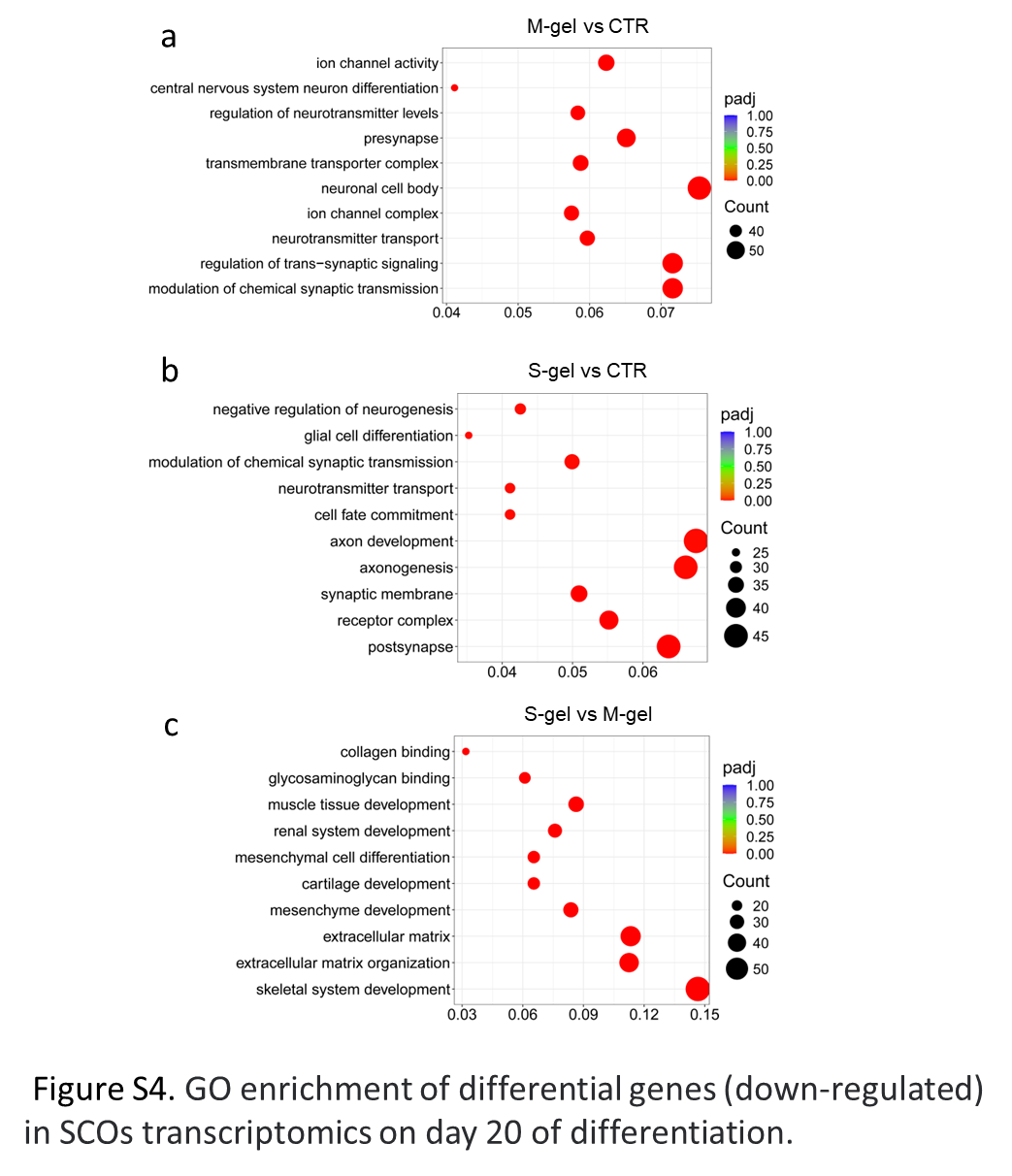
**

**
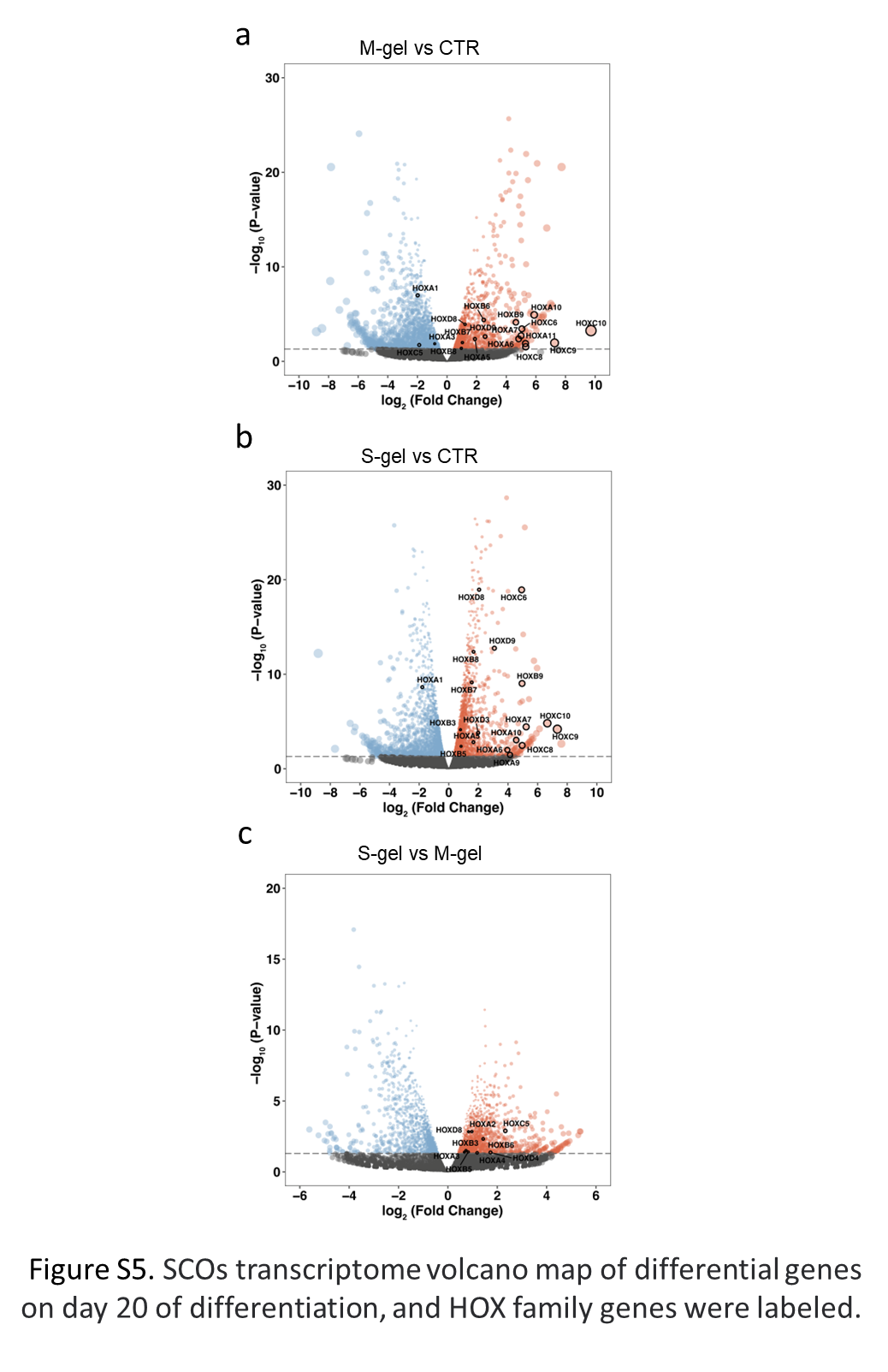
**

**
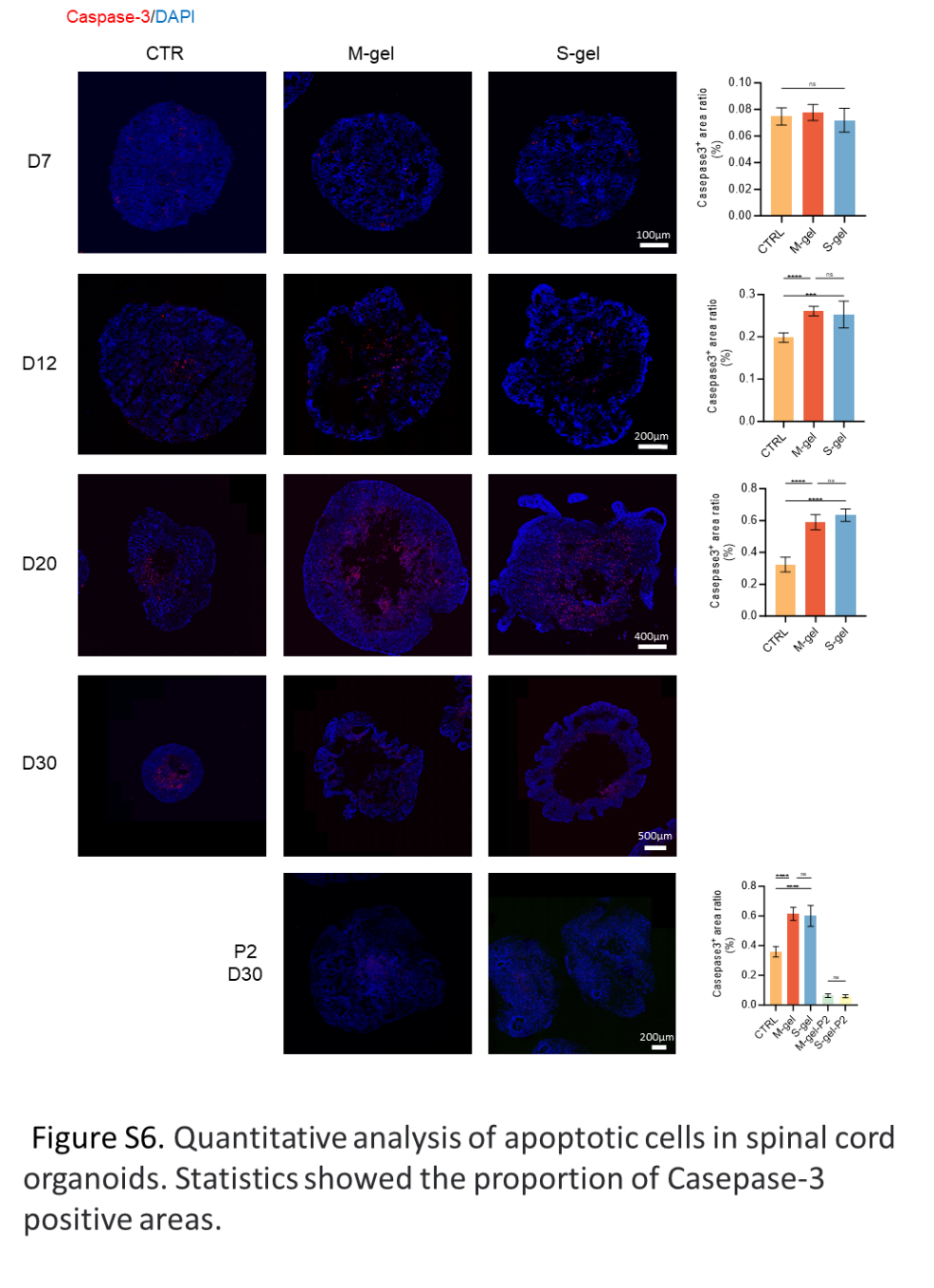
**

**
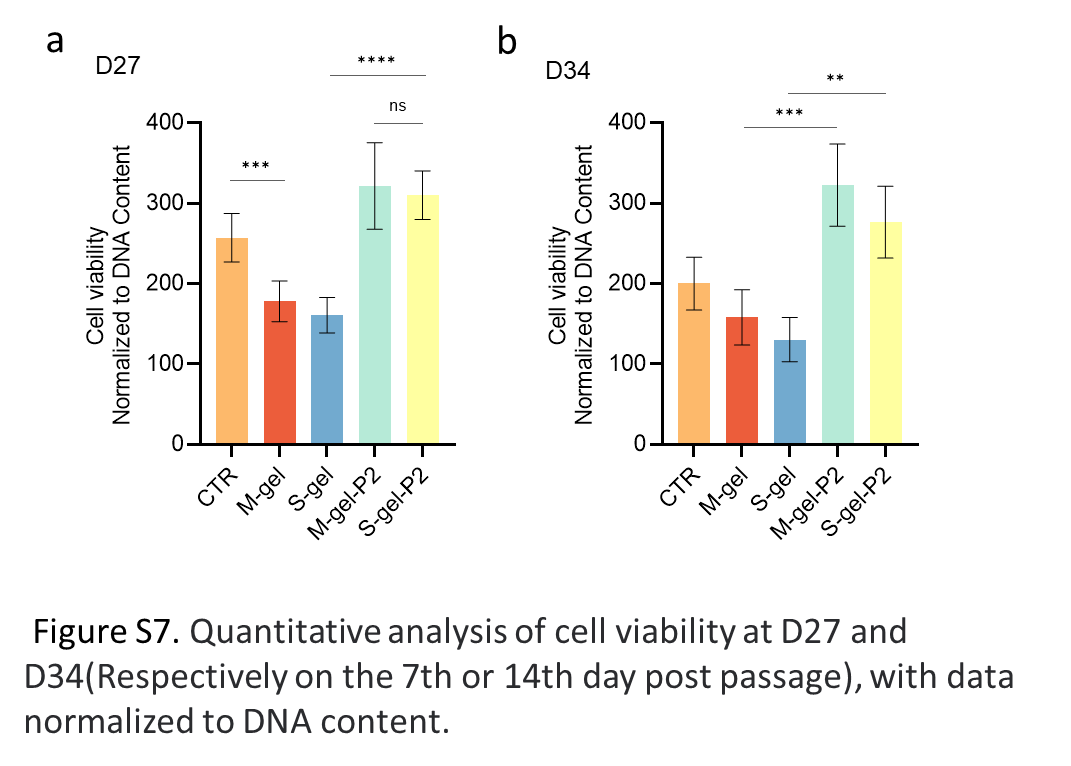
**
